# Blood Injury and Injection Phobia: The Neglected One

**DOI:** 10.1155/2014/471340

**Published:** 2014-06-24

**Authors:** Ab Latif Wani, Anjum Ara, Sajad Ahmad Bhat

**Affiliations:** Section of Genetics, Department of Zoology, Faculty of Life Science, Aligarh Muslim University, Aligarh, Uttar Pradesh 202002, India

## Abstract

Blood injury and injection (BII) phobia is a unique phobia associated with a diphasic cardiovascular response. The aim of this survey was to report the prevalence of BII phobia, its heritability, and clinical characteristics among the males and females in the Indian subcontinent. An interview and a survey were conducted using a developed BII phobia 21-item questionnaire among 3261 participant males (*n* = 1648) and females (*n* = 1613). Cronbach' alpha (*α*) of 0.972 of internal consistency was reported. The prevalence of BII phobia and associated fainting in females was slightly more than double in the males with a significant gender related effect. Similar avoidance behaviours involving hospital visits were reported for both males and females. The relative frequency of BII phobia among first and third degree relatives was found to be higher than among second degree relatives. Depression was found highly comorbid with BII phobia while a low rate of obsessive compulsion disorder (OCD) and social anxiety disorder (SAD) was reported. Morbidity associated with BII phobia may increase dramatically when other medical problems coincide with it.

## 1. Introduction

Blood injury injection (BII) phobia is a common psychiatric disorder, with an estimated prevalence of 3% to 4% in the general population [1, 2]. While most patients with blood injury phobia will not look for medical assistance and refuse medical appointments because of anxiety symptoms emerging with exposure, do not present to clinics or hospitals, and generally refuse hospital appointments because these things act as a phobic stimulus to them which increase their anxiety. BII phobia is a condition in which people are likely to faint at the sight of blood, the anticipation of physical injury, or the anticipation of an injection, characterized by avoidance behavior and intense, irrational fear in response to seeing blood, injections, injuries, disability, or exposure to these or other similar medical procedures [3–6]. It is often called a blood-injury-injection (BII) phobia because often blood, injury, and injection are the cues that can trigger a faint as is generally represented in the literature as BII phobia. As modern medicine mostly depends on injections, BII phobia has become an increasingly important issue. “Blood-injury phobia” is a curious type of specific phobia with distinct clinical features. Exposure to phobic cues induces tachycardia in most phobias. Blood-injury phobia patients typically experience a diphasic cardiovascular response of an initial tachycardia, followed by bradycardia, hypotension, shock, vertigo, syncope, diaphoresis, nausea, and seldom asystole and death [7, 8]. In 80% of the cases, the phobia response is characterized by syncope or presyncope [9, 10]. This response is peculiar to blood phobia and is not a characteristic of other specific phobias. Disgust levels in BII phobia were found to be more intense for the stimuli relative to fear levels [11].

Blood-injury phobia is very different from other specific phobias as the association of BII phobia with fainting is not common to other phobias [12, 13]. There are some physiological changes which they share with other phobias and anxiety disorders, for example, an increase in several hormones; the stress hormones, for example, have been reported to elevate during needle stimulation. Increased cortisol and corticotrophin (ACTH) level secondary to venipuncture and needle phobia have been documented [7, 14, 15].

A review of literature on fears and phobia shows that 21.2% of 449 Canadian women experienced mild to intense fear, and 4.9% had a phobic level of fear of injections, blood, injury, doctors, dentists, and hospitals [2]. Approximately 5% to 15% of the population, for example, decline necessary dental treatment, primarily because they fear oral injections [16].

Community survey by Fredrikson et al. found a point-prevalence of 3.05 for blood-injury injection phobia in Sweden [17, 18]. Patients with blood-injury-injection phobia may avoid hospitals, insulin injections for diabetes, or other important medical procedures. Noyes Jr. et al. reported that almost one quarter of persons with at least one illness/injury had much more nervousness than those without any illness [19]. About 11% of the subjects with dental anxiety and subtypes of BII phobia, respectively, reported high probability of avoiding dental treatment in a situation where a dental injection was possibly needed [20].

The devastating effects of BII phobia as an incapacitating mental illness in itself as well as a source of avoidance to modern medicine are prevalent in middle eastern cultures. It is a fact that there are not any reports of earlier studies on blood injection and injury phobia in the Indian subcontinent. Moreover it is pretty clear that in India there are millions of residents who have not visited hospitals due to a poor belief in traditional medicine. Some percentage may actually be reported by BII phobics. We performed the first survey study to describe the impact the BII phobia in Indian population.

## 2. Methodology

A survey was performed in the Aligarh region which is located at the coordinates 27.88°N and 78.08°E and is the part of a northern Indian state of Utter Pradesh. This survey includes subjects from its five prominent places: (1) Maulana Azad Nagar, (2) Hamdard Nagar and its adjacent areas, (3) Logo Colony and its adjacent areas (4), Firdous Nagar, and (5) Dhourra Mafi. The data recollected during this survey by this study was classified among the population included and subdivided into 5 different samples for further analysis.

Total of 3261 subjects from general population were interviewed with a semistructured based questionnaire for blood, injection, and injury phobia. The Cronbach's *α* value reported were 0.972 revealing high internal consistency among the items included in the questionnaire.

In order to confirm BII phobia diagnosis, probands were selected and the questionnaire based on DSM-V (APA, 2013) diagnostic criteria administered to patients. Additionally, probands and all available first, second, and third degree relatives were personally interviewed using a selected format questionnaire. Moreover, every proband and his or her sufferer relative was interviewed. Subsequently, pictures, paintings, or photographs of things and situations of which they were found to be afraid of were displayed upon them (e.g., pictures of injured people and bleeding wounds were shown to the respondents of blood phobia). This was performed in order to know whether the patient is having subthreshold fear or if a specific BII phobia has developed. Thus these pictorial stimuli served as conditional stimuli to provoke an irrational cognitive fear or autonomic arousal (sweating, pale faces, shivering, increased heart beats, fast pulse rate, and deep heavy breathings, etc.) in true phobic patients for further testing.

Furthermore, an assessment of family history, life history, and pedigree analysis calculating the phenotypic ratio between sufferer and nonsufferer relative of probands was performed. The penetrance of BII phobia in the sibs of I, II, and III degree relatives of probands was also measured by thorough analysis of pedigrees.

A number of subjects reported having more than two types of phobias or a phobic condition; these specific complex cases were covered under the comorbid status of the survey.

### 2.1. Statistical Analysis

In this study, standard statistical tests including Cronbach's *α* and one-way ANOVA were performed, to determine where true differences were present between the samples.

As stated above Cronbach's *α* for the BII phobia questionnaire was calculated to be 0.972. The principal component analysis of the BII phobia questionnaire was conducted using principal axis factoring and varimax rotation. SPSS program for windows release 16.0 (SPSS Inc., Chicago, IL, USA) was used for all the statistical analysis.

## 3. Results

Random survey samples collected from different places of Aligarh region involving a composite of 3261 individuals were obtained. Out of these, 1648 (50.53%) were male and 1613 (49.46%) were female subjects. Males suffering from BII phobias were 196 (mean 11.19%, SD ± 5.35) while females had a diagnosis of BII phobia in 384 of the cases (23.36%, SD ± 8.18) (Table 1). A significant higher percentage of females compared to males had BII phobia. This difference remains to be significant with all five samples. An overall mean percentage of affected (17.78%, SD ± 4.17) and nonaffected subjects (82.16%, SD ± 4.17) with BII phobia was also calculated.

Frequency of BII phobia in the first, second, and third degree relatives of probands was compared. The first degree relatives (parents, children, and sibs) showed the highest percentage of 26.42% (SD ± 8.59) followed by third degree relatives represented by grandparents, cousins, nieces, and nephews, whose percentage was 20.91% (SD ± 7.74). Interestingly, the second degree relatives (uncle, aunt, etc.) were affected by BII phobia in a very low nonsignificant percentage of 8.20% (SD ± 8.56) compared to the first and third degree relatives. Results are summarized in Table 2.

Although fainting is one of the specific phenomenological characteristics in BII phobia, our data shows the presence of both fainting and nonfainting subjects suffering from BII phobia. The mean prevalence of fainters from our data was calculated to be 55.77% (SD ± 4.30). A high percentage of females involving approximately 3/4 of affected individuals with BII phobia are significantly higher than that reported in males with BII phobia. The percent mean value for fainting in males suffering from BII phobia was 39.40% (SD ± 8.94), while the percent mean value for female sufferers was 64.09 (SD ± 6.49). Finally nonfainting subjects affected by BII phobia were represented in 44.22% (SD ± 4.30) of the sample. These results are illustrated in Table 3.

Comorbidity in psychiatry is generally a rule rather than an exception [21]. In BII phobia, comorbidity is highly prevalent [18, 21]. In our sample individuals with positive pedigrees for BII phobia, we also performed an analysis to explore possible psychiatric comorbidities. Other psychoneurotic disorders such as agoraphobia, depression, animal phobia, panic phobia, social anxiety disorder, and OCD are presented in Table 4.

The most common types of psychoneurotic disorders comorbid with BII phobia were agoraphobia (15.71% SD ± 5.40), animal phobia 13.17% (SD ± 2.76), and panic disorder 6.13% (SD ± 2.15). Thus the prevalence of social anxiety disorder (SAD) 5.17% (SD ± 1.28) and obsessive-compulsion disorder (OCD) with its percentage of mean value calculated as 2.57% (SD ± 2.49) was significantly lower. Not surprisingly, a depressive disorder (20.29%, SD ± 3.77) was the most common comorbidity with BII phobia in our sample. At the end of our analysis, 34.73% of BII phobia patients were reported as not suffering from any kind of measurable psychiatric comorbidity screened by the questionnaire.

The average age of onset was calculated to be 9.3 years (SD ± 3.27) for males and 7.5 years (SD ± 2.51) for females. There was a significant variation in data among the subjects depending on the age of BII phobia. First of all, the distribution was highly skewed. Secondly, very few BII phobia patients had onset at older ages, and maximum number of subjects never reported his or her phobia as a problem for which he or she sought mental treatment before. Therefore, only 5.3% of BII phobia patients reported to have visited the hospital for this specific problem once or twice without engaging in any kind of treatment. Thus 94.7% of the subjects with BII phobia had never visited physician or other health professionals for this phobia.

It is of interest that during our survey programme many of the subjects either did not want to share their phobia behaviours or hid from us how their lives changed related to their phobia. This may be related to irrational fears involving medical treatment [22].

## 4. Discussion

Psychiatric disorders exert a heavy burden on health care in modern societies. Understanding the genetic and environmental influences on the manifestation of phobic behaviors is important from the dual perspectives of human health and evolutionary psychobiology. Development of effective treatments depends on understanding the genetic mechanisms and environmental triggers that predispose to these disorders. Blood injury and injection (BII) phobia deals with the fear of injury, injection/needles, and blood [3]. BII phobia is most commonly diagnosed after a person faints, experiences a change in heart rate, or has extremely uncomfortable sensations around the sight of blood [3]. Most of the cases involving BII phobia are discovered in early childhood and require some method of treatment to overcome dysfunctional behaviours associated with adverse medical outcomes [23].

BII phobia is considered in some cases to have severe and may even become life threatening when essential medical procedures such as urgent surgery, blood transfusion, or insulin injections cannot be delivered. BII phobia patients avoid close contact with sick people, refuse hospital appointments, and would not watch television or read newspaper reports about trauma or disasters. It has been reported that women suffering from severe BII phobia may avoid pregnancy due to the blood and medical procedures involving childbirth [24].

In our sample survey, an important gender effect in the prevalence of BII phobia between males and females was reported. Rate of BII phobia Prevalence in females was 23.86% (±SD 8.18) significantly higher than males 11.19% (±SD 5.35). Specific phobias in general are twice as common in women as in men [25, 26]. Our results are within the trends reported for specific phobias in other samples from different geographical areas and cultural contexts [1, 25–30].

Blood and injection phobia is equally distributed in men and women in some studies while in others there is a trend for a female preponderance [1, 30–35]. In our survey a significantly higher prevalence of BII phobia was found in females than in males. Females were also earlier in onset of BII phobia than in males. The early age of onset reported in this survey is consistent with those previously reported for clinical samples of blood phobia [36] and ECA follow-up study [18]. On the other hand, the subjects of BII phobia in our survey had a greater proportion of histories of fainting (55.77%) compared to (25%) those in ECA follow-up study at Baltimore [18] but lesser than (77%) those in the clinical sample of blood and injection phobia [36]. There is a substantial body of evidence demonstrating a gender effect involving risk factors, incidence, age-at-onset, course, response to therapy, and underlying neurobiology of many common psychiatric disorders [37–40].

Our studies also show the significant differences in the relative frequencies of BII phobia among the first, second, and third degree relatives. Therefore multiple or quantitative genes can be partly responsible for some of these phobic traits, not following any sequential pattern for their transmission. This may perhaps also be due to variable environmental experiences. As gene-environment interaction refers to genotypic differences in susceptibility to an environmental exposure [41, 42]. The impact of sex on genetic risk may differ across phobia subtypes. Sex-specific genetic risk factors may exist for agoraphobia, social, situational, and blood-injury phobias but not for animal fear phobia [43].

BII phobia is also associated with many medical disorders, like cancer, diabetes, and cardiovascular disease [18, 44]; however, this survey includes only psychiatric comorbidities and it is a limitation to this study that we have not surveyed medical comorbidity with BII phobia. In evaluating the results of this survey, one more limitation needs to be considered, that the findings may not be the representative of the general population because of the sample size. However, this survey represents an estimation of phobic patients who are likely to avoid treatment.

## 5. Conclusion

BII phobia is a common phobia and is prevalent in females as compared to males. Though it is not so dreadful at its first place, it proves to be dangerous when present with other medical disorders. A large number of BII phobic patients are found to have never consulted a physician for treatment. It is recommended that health professionals must take initiative to keep all the BII patients on record and help them for the treatment. This deserves further study. 

*Screening Questionnaire for Blood/Injection/Injury Phobia*

- Name of the person
- Age of the person
- Sex of the person
- Standard of the school
- Occupation of the person (if employed)
- Address.

*The following Are the List of Items and Their Corresponding Factors*

1. Are you phobic of blood, injection, injury, and needle?  (Factor: 0.923)
2. Do you avoid seeing others' blood?  (Factor: 0.839)
3. Do you avoid looking at your own blood?   (Factor: 0.829)
4. What is the age of onset of your blood phobia? (Factor: 0.852)
5. Do you faint at the sight of blood?  (Factor: 0.721)
6. At what age did you start fainting at the sight of blood?  (Factor: 0.819)
7. Do you avoid receiving injections?  (Factor: 0.821)
8. Does needle size frighten you?  (Factor: 0.852)
9. Do you generally avoid gatherings and crowded places?  (Factor: 0.627)
10. Do you generally feel that heart rate is increased in phobic situations?  (Factor: 0.831)
11. Do you feel suffocation because of fear?  (Factor: 0.827)
12. Do you feel so scared that your tongue gets dried up?  (Factor: 0.852)
13. Do you feel that you lose presence of mind in phobic conditions?  (Factor: 0.752)
14. Do you feel chest pain in phobic conditions?  (Factor: 0.672)
15. Do you feel physical weakness?  (Factor: 0.679)
16. Do you feel hot flushes in phobic conditions?  (Factor: 0.856)
17. Do you feel that your mind is in numbness if you see serious objects?  (Factor: 0.721)
18. Do you feel difficulty in respiration in phobic conditions?  (Factor: 0.839)
19. Do you get disturbed by imagining the phobic conditions or objects?  (Factor: 0.721)
20. Do you feel shortness of breath in phobic conditions?  (Factor: 0.852)
21. Do you feel sweat in phobic conditions?  (Factor: 0.771)
22. Do you feel lack of sleep by imagining the phobic conditions?  (Factor: 0.711)

(Extraction method: principal component analysis)

## Figures and Tables

**Table 1:** Showing the gender wise differences in the percentage of patients suffering from BII phobia in five localities of Aligarh.

Five different samples	Males				Females			
	Total	BII phobics	BII phobics %	*F* value and *P* value	Total	BII phobics	BII phobics %	*F* value and *P* value
Sample 1	282	34	12.05 ± 5.0	*P* < 0.05 *F* = 1.27	253	62	24.5 ± 9.49	*P* < 0.05 *F* = 2.15
Sample 2	327	44	13.45 ± 6.12		293	72	24.57 ± 8.25	
Sample 3	455	55	12.0 ± 7.41		450	115	25.55 ± 9.01	
Sample 4	238	29	12.18 ± 4.07		221	53	23.98 ± 7.52	
Sample 5	346	34	9.82 ± 4.11		396	82	20.7 ± 9.82	
	1648	196	% Mean ± SD = 11.19 ± 5.35, SEM = ±1.585,		1613	384	% Mean ± SD = 23.86 ± 8.18, SEM = ±1.724	

**Table 2:** Showing the relative frequencies of blood injection and injury phobia among the first, second, and third degree relatives of probands.

Five different samples	First degree relatives			Second degree relatives			Third degree relatives		
	Total	Sufferers	Sufferer % ± SD	Total	Sufferers	Sufferer % ± SD	Total	Sufferers	Sufferer % ± SD
Sample 1	188	53	28.19 ± 9.3	180	9	5.00 ± 7.39	113	24	21.23 ± 6.67
Sample 2	236	58	24.57 ± 10.1	233	22	9.44 ± 9.0	136	34	25.0 ± 7.01
Sample 3	330	94	28.48 ± 8.19	321	31	9.65 ± 9.0	181	38	20.99 ± 6.61
Sample 4	167	45	26.94 ± 8.33	171	18	10.52 ± 8.8	99	20	20.2 ± 8.06
Sample 5	255	61	23.92 ± 7.0	187	12	6.41 ± 8.55	169	29	17.15 ± 10.36

	% Mean ± SD = 26.42 ± 8.59, *P* < 0.03, *F* = 2.29			% Mean ± SD = 8.20 ± 8.56, *P* < 0.05, *F* = 3.21			% Mean ± SD = 20.91 ± 7.74, *P* < 0.03, *F* = 2.61		

**Table 3:** Showing the gender wise differences in the percentage of fainting and nonfainting sufferers for BII phobia in different samples of the population.

Five different samples	Percentage of total fainter	Percentage of total nonfainter	Percentage of total fainter males	Percentage of total nonfainter males	Percentage of total fainter females	Percentage of total nonfainter females	(O.R)	95% CI
Sample 1	57.14	42.86	28.57	71.43	66.66	33.34	0.42	0.07, 2.37
Sample 2	54.54	45.46	38.46	61.54	65.0	35.0	0.59	1.17, 2.05
Sample 3	53.33	46.67	50.0	50.0	62.06	37.94	0.80	0.28, 2.26
Sample 4	62.50	37.5	33.33	66.67	72.22	27.78	0.46	0.08, 2.66
Sample 5	51.35	48.65	46.66	53.34	54.54	45.45	0.85	0.27, 2.67

	Mean ± SD 55.77 ± 4.30	Mean ± SD 44.22 ± 4.30	Mean ± SD 39.40 ± 8.94	Mean ± SD 60.59 ± 8.94	Mean ± SD 64.09 ± 6.49	Mean ± SD 35.90 ± 6.49	Mean (O.R) 0.66	0.37, 1.16

The percentage of fainting among male and female subjects in the cases of BII phobia in males as 25.57%, SD ± 4.46, and in females as 74.41%, SD ± 4.46.

“O.R” is odds ratio; CI is confidence interval; *P* < 0.05.

**Table 4:** Showing different types of comorbidities with blood injection and injury phobia in different samples of the population.

Five different samples	Depression			Agoraphobia			Animal phobia			Panic disorder			Social anxiety disorder			OCD		
	%C	O.R	95% CI	%C	O.R	95% CI	%C	O.R	95% CI	%C	O.R	95% CI	%C	O.R	95% CI	%C	O.R	95% CI
Sample 1	17.85	2.40	0.31, 8.55	17.85	2.40	0.31, 18.55	10.71	1.58	0.12, 20.68	7.14	3.33	0.18, 61.68	3.57	0.91	0.03, 24.90	—	—	—
Sample 2	18.18	0.72	0.11, 4.68	9.09	0.75	0.06, 9.22	12.12	0.47	0.04, 5.10	9.09	0.75	0.06, 9.22	6.06	1.58	0.09, 27.77	3.03	0.48	0.01, 12.73
Sample 3	24.44	1.04	0.25, 4.31	20.00	0.44	0.08, 2.47	15.55	1.44	0.27, 7.43	8.88	1.92	0.24, 15.18	6.66	0.90	0.07, 10.76	4.44	1.86	0.10, 32.01
Sample 4	16.66	4.0	0.42, 37.77	20.83	0.85	0.07, 9.44	16.66	1.13	0.08, 11.93	4.16	1.06	0.03, 24.94	4.16	0.89	0.03, 24.94	—	—	—
Sample 5	24.32	0.66	0.13, 3.22	10.81	0.50	0.04, 4.82	10.81	1.53	0.19, 12.32	5.40	1.50	0.08, 26.01	5.40	1.50	0.08, 26.01	5.40	1.50	0.08, 26.01

Overall % mean ± SD	20.29 ± 3.77			15.71 ± 5.40			13.17 ± 2.76			6.13 ± 2.15			5.17 ± 1.28			2.57 ± 2.49		

“%C” indicates percentage of comorbidity, “O.R” is odds ratio, “CI” is confidence interval, and “OCD” is obsessive compulsive disorder.
